# Xylooligosaccharides Increase *Bifidobacteria* and *Lachnospiraceae* in Mice on a High-Fat Diet, with a Concomitant Increase in Short-Chain Fatty Acids, Especially Butyric Acid

**DOI:** 10.1021/acs.jafc.0c06279

**Published:** 2021-03-16

**Authors:** Karin Berger, Stephen Burleigh, Maria Lindahl, Abhishek Bhattacharya, Prachiti Patil, Henrik Stålbrand, Eva Nordberg Karlsson, Frida Hållenius, Margareta Nyman, Patrick Adlercreutz

**Affiliations:** † Department of Experimental Medical Science, Lund University, P.O. Box 188, SE-221 00 Lund, Sweden; ‡ Division of Biotechnology, Department of Chemistry, Lund University, P.O. Box 124, SE-221 00 Lund, Sweden; § Division of Biochemistry and Structural Biology, Department of Chemistry, Lund University, P.O. Box 124, SE-221 00 Lund, Sweden; ∥ Department of Food Technology, Engineering and Nutrition, Lund University, P.O. Box 124, SE-221 00 Lund, Sweden

**Keywords:** prebiotics, xylooligosaccharides, Bifidobacteria, Lachnospiraceae, S24-7, short-chain fatty acids, butyrate, high-fat diet

## Abstract

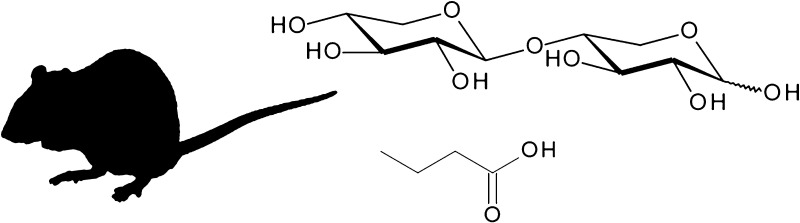

Effects of xylooligosaccharides (XOSs) as well as a mixture of XOS, inulin, oligofructose, and partially hydrolyzed guar gum (MIX) in mice fed a high-fat diet (HFD) were studied. Control groups were fed an HFD or a low-fat diet. Special attention was paid to the cecal composition of the gut microbiota and formation of short-chain fatty acids, but metabolic parameters were also documented. The XOS group had significantly higher cecum levels of acetic, propionic, and butyric acids than the HFD group, and the butyric acid content was higher in the XOS than in the MIX group. The cecum microbiota of the XOS group contained more *Bifidobacteria*, *Lachnospiraceae,* and S24-7 bacteria than the HFD group. A tendency of lower body weight gain was observed on comparing the XOS and HFD groups. In conclusion, the XOS was shown to be a promising prebiotic candidate. The fiber diversity in the MIX diet did not provide any advantages compared to the XOS diet.

## Introduction

There is increasing evidence that the gut microbiota influences the health of animals and humans to a large extent. An attractive way toward a health promoting gut microbiota is to design the diet so that it promotes the desired microbes. Prebiotics is defined as substances which selectively stimulate the growth of beneficial microbes, thereby providing health benefits. Not surprisingly, the search for efficient prebiotics is a very active research area at present.^1−3^

Fructose-based prebiotics are quite well established, with long-chain inulin and shorter fructo-oligosaccharides as main representatives. Furthermore, galacto-oligosaccharides, often produced from lactose, are widely used as prebiotics in foods, such as dairy products. Guar gum has often been associated with beneficial metabolic effects such as decreasing serum blood lipids and lower postprandial blood glucose levels after a meal, which often has been attributed to its viscous properties. Another mechanism would be that guar gum is highly degraded by the colon microbiota, giving rise to high amounts of especially propionic acid.^4,5^

Arabinoxylan, xylo-oligosaccharides (XOSs), and arabinoxylo-oligosaccharides constitute less thoroughly researched alternatives, which have been shown to have prebiotic properties.^6−8^ These products are typically produced from hemicellulose-rich industrial side streams. They are present as natural components in several foods and are generally regarded as safe (GRAS) by FDA https://www.accessdata.fda.gov/scripts/fdcc/index.cfm?set=GRASNotices&id=458&sort=Substance&order=ASC&startrow=1&type=basic&search=458. Furthermore, a European Food Safety Authority (EFSA) panel found XOS safe for use.^9^

We have previously studied potential prebiotic effects of cereal by-products. A product prepared from rye bran, and with XOS as a main constituent, caused a significant increase in *Bifidobacteria* in the cecum of mice on a high-fat diet along with indications of improved metabolic function and increased production of propionic acid.^10^ To investigate if these effects indeed were due to XOS, the present study was carried out using pure XOS as a supplement in the same animal model, and the dose was somewhat increased to 8% (w/w) to get clearer effects. In addition, a more diverse supplement, containing XOS and more established prebiotics based on fructose and partially hydrolyzed guar gum (MIX), was tested. Equal amounts of XOS, fructose-based prebiotics and partially hydrolyzed guar gum were used in the MIX diet, with a total amount of 8% (w/w). The fructose-based product was a mixture of equal amounts of inulin and oligofructose. Both a high-fat diet (HFD) and a low-fat diet (LFD) were used as controls. Active fermentation of the supplemented test compounds was expected to occur in the cecum and therefore special attention was paid to the composition of the cecal microbiota and the formation of short-chain fatty acids (SCFAs).

## Materials and Methods

### Test Products

Inulin (Orafti GR) and Oligofructose (Orafti P95) were gifts from Alsiano A/S, Birkeröd, Denmark. Xylooligosaccharides (XOS95P) were purchased from Shandong Longlive Bio-Tech Co., Ltd, Shandong, China. The dominating oligosaccharides were xylobiose, xylotriose, and xylotetraose (in total 78% (w/w)). Meritene, a product containing partially hydrolyzed guar gum (galactomannan), was produced by Nestlé Health Science and purchased from a local pharmacy. The control HFD and LFD contained 8% cellulose BW200 (calculated on dry weight basis). The compositions of these four commercial products are shown in Table 1.

**Table 1:** Composition in % (w/w) of Test Products Used in the Various Diets

	inulin (Orafti GR)	oligofructose (Orafti P95)	XOS95P	Meritene
dietary fiber	90.1	92	96	86
glucose, fructose, sucrose	7.1	4.9		
sugar				6
ash			0.3	
moisture	2.8	3.1	2.3	

### Diets

The groups HFD, XOS, and MIX got a high-fat diet with 60 energy % from fat (lard as dominating the energy source), while the LFD group got 11 energy % from fat (wheat starch as the dominating energy source). The diet of the XOS group contained 8% (w/w) XOS95P, while the MIX group received a diet containing 2.7% XOS95P, 2.7% Meritene, 1.3% Orafti P95, and 1.3 %Orafti GR. The diets are described fully in the Supporting Information (Table S1).

### Mouse Study

#### Animals and Study Design

Male 5-week-old C57BL/6J BomTac mice (Taconic, Skensved, Denmark) were delivered and housed four mice per cage. During acclimatization, they were all fed an LFD for 8 days. The animals were maintained in a temperature-controlled room with a 12 h light–dark cycle. All animal procedures were approved by the Malmö/Lund Ethical Committee for Animal Experiment (Approval M10-15, Lund, Sweden) and were carried out in accordance with the relevant guidelines. After acclimatization, the mice were either fed one of the control diets (HFD or LFD) containing cellulose as the fiber source or one of the two experimental diets in which the cellulose was substituted for XOS or MIX (details on the diets are in Table S1). The mice were fed the different diets *ad libitum* with free access to drinking water for 9 weeks. Body weight and food intake were registered once a week. The energy intake was calculated based on registered food consumption. At the time of sacrifice, mice were fasted for 4 h and thereafter blood was drawn from the vena saphena followed by cervical dislocation. Serum was prepared by centrifugation of blood samples and stored at −80 °C until analysis. Body fat content and lean body mass were analyzed using dual-energy X-ray absorptiometry (DEXA) technique with a Lunar PIXImus densitometer (GE Medical Systems). The epididymal adipose tissue and the cecum were thoroughly excised and weighed. The cecal content was collected and snap-frozen for SCFA analysis and the cecum tissues (walls) were rinsed in sterile phosphate buffered saline and weighed (liquid was thoroughly soaked up by sterile compress) and tissues were snap-frozen. All samples were stored at −80 °C until analysis.

#### Serum Analysis and Assessment of Insulin Resistance

Blood glucose levels were immediately measured using a glucometer (Onetouch Ultra2; Lifescan, Milpitas, CA, USA). Insulin, serum amyloid A (SAA), and LPS-binding protein (LBP) were measured in plasma using commercial ELISA kits (Mercodia, Uppsala, Sweden, Tridelta Development Ltd, Wicklow, Ireland and Nordic Biosite, Täby, Sweden, respectively).

### Analysis of SCFAs

SCFAs were analyzed by gas–liquid chromatography according to Zhao et al. 2006.^11^ The frozen content from the cecum was thawed (1 g), suspended in water, and homogenized (3 min). The pH of the suspension was adjusted to approximately 2 with 5 M HCl and then the samples were shaken (10 min) and centrifuged (20 min, 5000 rpm) to get a clear supernatant. The internal standard, 2-ethylbutyric acid, was added into the supernatant to give a final concentration of 1 mM in the sample and was then injected in the GC for analysis.

### DNA Extraction, PCR Amplification, and Sequencing

DNA from cecum microbiota was extracted using the QIAamp Power Fecal DNA Kit (Qiagen, Hilden, Germany) according to the manufacturer’s instructions. Measurement of DNA concentration was performed using a Fluoroskan fluorometer (Thermo Fisher Scientific). The V3–V4 region of 16S rRNA genes was amplified using forward and reverse primers containing Illumina overhang adaptors and unique dual indexes. The sequence of the 16S amplicon primers were (Forward)-5′ TCGTCGGCAGCGTCAGATGTGTATAAGAGACAGCCTACGGGNGGCWGCAG and (Reverse)-5′ GTCTCGTGGGCTCGGAGATGTGTATAAGAGACAGGACTACHVGGGTATCTAATCC following Klindworth et al.^12^ Paired-end sequencing with a read length of 2 × 250 bp was carried out on a Miseq Instrument (Illumina, San Diago, USA) using a Nextera XT Index Kit (Illumina, San Diago, USA). As an internal control, 5% of PhiX was added to the amplicon pool. Illumina sequencing adaptors were trimmed off during the generation of FASTQ files and reads that did not match any barcodes were discarded.

### Sequence Analysis

Sequence data were analyzed with the open-source bioinformatics pipeline Quantitative Insights Into Microbial Ecology (QIIME).^13^ Sequences were removed when lengths were <200 nucleotides, >290 nucleotides, or when the quality score fell below 25 as determined using PRINSEQ software.^14^ After filtering, a total of 3,988,987 reads were obtained from 45 samples with an average of 88,642 reads per sample (min: 37,019 and max: 119,305). The sequences were normalized by rarefaction (depth of 52,880) using Qiime, whereby one sample fell below this cut-off and was therefore excluded from the experiment. The remaining samples were grouped into operational taxonomic units (OTUs) at a minimum of 97% similarity by using Qiime’s closed reference method based on the Greengenes database (v.13.8) and filtered by the removal of singletons and low abundance OTUs (minimum count fraction set at 0.001).

### Statistics

#### Mouse Study

All groups were compared to the HFD control. Body weight gain and food intake were analyzed with two-way analysis of variance (ANOVA) with Dunnet’s multiple comparison post-test since the data were Gaussian-distributed according to the D’Agostino and Pearson normality test. When not normally distributed, Kruskal–Wallis non-parametric test with Dunn’s multiple comparison post-test were used. (GraphPad Prism 8.2, GraphPad Software, San Diego, CA, USA).

#### Sequence Analysis

A Qiime-based permanova (using the pseudo-F statistical test and 999 permutations) was used to test for overall differences between the microbiomes in the four treatments, while a Qiime-based heat map (MetaPhlan) was used to visualize relative differences in the microbiomes at the family and genus levels. Bar charts and box plots were created and statistics calculated using GraphPad Prism (8.4.2.), whereby the data were tested for normal distribution using the Shapiro–Wilk test (α = 0.05). If normal, an ANOVA was performed; otherwise the data were considered non-parametric and a Kruskal–Wallis Rank Sum Test was performed (α = 0.05). Pairwise comparisons (Fisher’s Least Significant Difference if normal, otherwise a Wilcoxon Rank Sum Test) were carried out using R (3.6.0).

A partial least squares (PLS-X) loading variable and score scatter plot (bi-plot) were carried out using SIMCA software (version 15.0.2, Umetrics, Sartorius Stedim Data Analytics AB Sweden). The loading variables were the treatments, biomarkers, and microbiome data, while the scores were the observations of the four treatments. Estimates of the *Bifidobacterium* species’ relative abundance in each of the four treatments was calculated as follows: All OTUs corresponding to *Bifidobacterium* were identified in each treatment and their associated reads collected. A subset of reads (*n* = 100) from each of these groups was randomly, proportionally selected based on the relative abundance of their corresponding OTUs and blasted at NCBI (blastn, 16S database). The closest matching species was assigned to each read. The experiment was carried out six times and an ANOVA performed using Microsoft Excel to test if there were differences in the *Bifidobacterium* species’ relative abundance between the four treatments. The results were plotted as a stacked bar chart using ggplot2 (14).

## Results

### Physiological Observations

**Figure 1:**
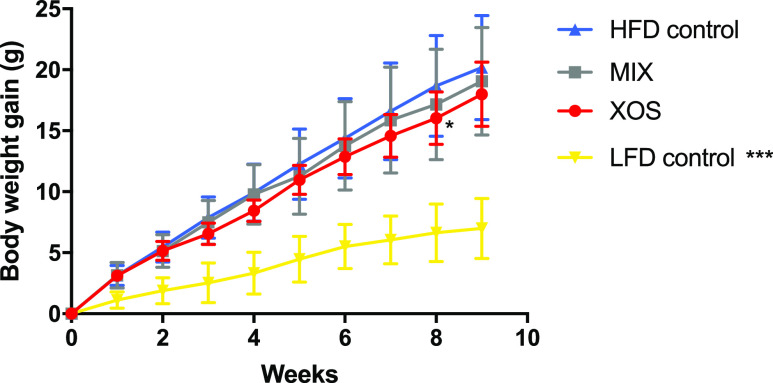
Weekly body weight registration. Mean ± SD. Statistical comparisons of body weight compared to the control were made using a two-way ANOVA with the Bonferroni post-test.

Body weight gain was significantly lower in the LFD group compared to the HFD control group from the second week to the end of the study. A tendency of lower body weight gain was observed between the XOS group and the HFD control in the last weeks of the study but was significantly different (*P* < 0.05) only at week eight (Figure 1). Food intake, calculated as energy intake per day, was significantly lower in the LFD group but no significant differences were observed between the three groups fed an HFD (Table 2). Body fat content, measured as fat tissue, using DEXA scan and weight of epididymal fat pads, showed significantly lower fat content in mice fed an LFD, while all HFD-fed groups were equal in adiposity. No difference in lean tissue body mass was observed between groups.

**Table 2:** Body Weight, Body Composition, and Plasma Parameters^a^

	HFD	LFD	MIX	XOS
body weight start (g)	20.8 ± 1.5	21.3 ± 1.6	21.4 ± 2.2	21.5 ± 1.9
body weight end (g)	40.9 ± 4.2	28.3 ± 2.1^d^	40.5 ± 5.3	39.45 ± 4.1
body weight gain (g)	20.2 ± 4.3	7.0 ± 2.5^d^	19.1 ± 4.4	18.0 ± 2.6
feed efficiency ratio (g weight gain/kcal)	0.024 ± 0.006	0.011 ± 0.007^d^	0.024 ± 0.007	0.022 ± 0.007
feed intake (g/mouse/day)	2.60 ± 0.11	2.74 ± 0.14	2.48 ± 0.13	2.52 ± 0.17
feed intake (kcal/mouse/day)	13.6 ± 0.61	10.1 ± 0.53^d^	12.7 ± 0.66^a^	12.8 ± 0.85
body fat (%)	36.8 ± 4.8	14.1 ± 2.6^d^	37.1 ± 2.9	33.4 ± 6.8
epididymal fat (g)	1.61 ± 0.34	0.31 ± 0.09^d^	1.79 ± 0.33	1.65 ± 0.50
cecum total (g)	0.25 ± 0.04	0.32 ± 0.05^a^	0.34 ± 0.06^c^	0.34 ± 0.08^c^
cecum content (g)	0.17 ± 0.04	0.23 ± 0.04^a^	0.23 ± 0.05^a^	0.23 ± 0.06^a^
cecum tissue (g)	0.08 ± 0.01	0.08 ± 0.01	0.11 ± 0.01^d^	0.11 ± 0.02^d^
blood glucose (mM)	11.1 ± 1.5	7.6 ± 1.4^d^	12.2 ± 2.1	10.3 ± 1.4
insulin (μg/L)	6.6 ± 5.2	0.76 ± 0.29^c^	6.5 ± 3.9	6.4 ± 3.6
LBP (μg/mL)	3.4 ± 0.58	4.1 ± 1.6	3.6 ± 0.27	4.3 ± 1.8
SAA (μg/mL)	19.2 ± 5.8	23.4 ± 12.6	22.3 ± 12.2	24.6 ± 28.6

a Mean ± SD. ^a^*p* < 0.05, ^b^*p* < 0.01, ^c^*p* < 0.001, ^d^*p* < 0.0001 compared to HFD.

Blood glucose control was measured as fasting glucose and insulin. Only LFD showed significantly lower blood glucose and insulin levels compared to the HFD control.

Cecum was excised and weighed both with (=total) and without (=tissue) cecal content. Both XOS and MIX significantly increased the total cecum and cecal tissue weight compared to the HFD control.

The inflammatory markers SAA and LBP did not differ between groups (Table 2).

### SCFAs in the Cecum

Acetic acid (32–54 μmol/g) was the main SCFA formed in the cecum of mice, followed by propionic- (5–10 μmol/g) and butyric acids (4–12 μmol/g), which corresponded to 69–74, 10.8–14.0, and 8.4–15.6% of the total amount of SCFAs formed, respectively (Table 3). Considerable amounts of valeric (0.8–1.0 μmol/g), iso-valeric (0.8–1.0 μmol/g), and iso-butyric acids (0.7–0.8 μmol/g) were also detected (1–2% of total SCFAs), while there were only minor amounts of caproic- and heptanoic acids (<0.05%).

**Table 3:** Concentrations (μmol/g) of SCFA in Cecum of Mice

	HFD	LFD	MIX	XOS
acetic	35.5 ± 5.4^b^	34.2 ± 7.5^b^	47.7 ± 17.5^ab^	52.7 ± 16.9^a^
propionic	5.3 ± 0.9^b^	6.0 ± 1.3^b^	9.6 ± 2.8^a^	9.3 ± 1.9^a^
iso-butyric	0.8 ± 0.1^a^	0.8 ± 0.2^a^	0.8 ± 0.2^a^	0.7 ± 0.2^a^
butyric	5.0 ± 1.2^bc^	3.9 ± 1.3^c^	7.7 ± 4.1^b^	11.9 ± 5.2^a^
iso-valeric	0.9 ± 0.1^a^	0.8 ± 0.1^a^	1.0 ± 0.3^a^	0.9 ± 0.2^a^
valeric	1.0 ± 0.2^a^	0.8 ± 0.2^b^	0.9 ± 0.1^ab^	1.0 ± 0.2^a^
caproic	0.0 ± 0.0^a^	0.0 ± 0.0^a^	0.0 ± 0.0^a^	0.0 ± 0.0^a^
heptanoic	0.0 ± 0.0^a^	0.0 ± 0.0^a^	0.0 ± 0.0^a^	0.0 ± 0.0^a^
total	48.6 ± 6.7	46.5 ± 9.4	67.8 ± 19.9	76.6 ± 22.0

a Values are means ± SEM, *n* = 10. Mean values in the same row with unlike superscript letters are significantly different. *P* < 0.05. 0.0 is less than 0.03 μmol/g.

The total and the individual concentrations of SCFAs were quite similar for the HFD and LFD control groups, indicating that the amount of fat and starch had a minor influence on the cecal SCFAs formed (Table 3). The cecal concentrations with these diets were also significantly lower than with the diets containing XOS and MIX concerning total SCFAs (mean 48.6 μmol/g for the HFD group vs 76.6 μmol/g and 67.8 μmol/g for groups fed XOS and MIX, respectively), acetic acid (mean 35.5 μmol/g vs 52.7 μmol/g, for XOS and 47.7 for MIX, respectively), propionic acid (mean 5.3 μmol/g vs mean 9.3 and 9.6 μmol/g for mice fed XOS and MIX, respectively), and butyric acid (mean 5.0 vs 11.9 μmol/g and 7.7 μmol/g in mice fed XOS and MIX, respectively). Concerning butyric acid, the concentration for the group fed XOS was significantly (*P* < 0.05) higher than for the group fed MIX.

The distribution of SCFAs in the control groups (LFD and HFD) was also very similar. XOS gave a higher proportion of butyric acid than groups fed the other diets, while the group fed MIX gave a higher proportion of propionic acid.

### Microbiota Analysis

**Figure 2:**
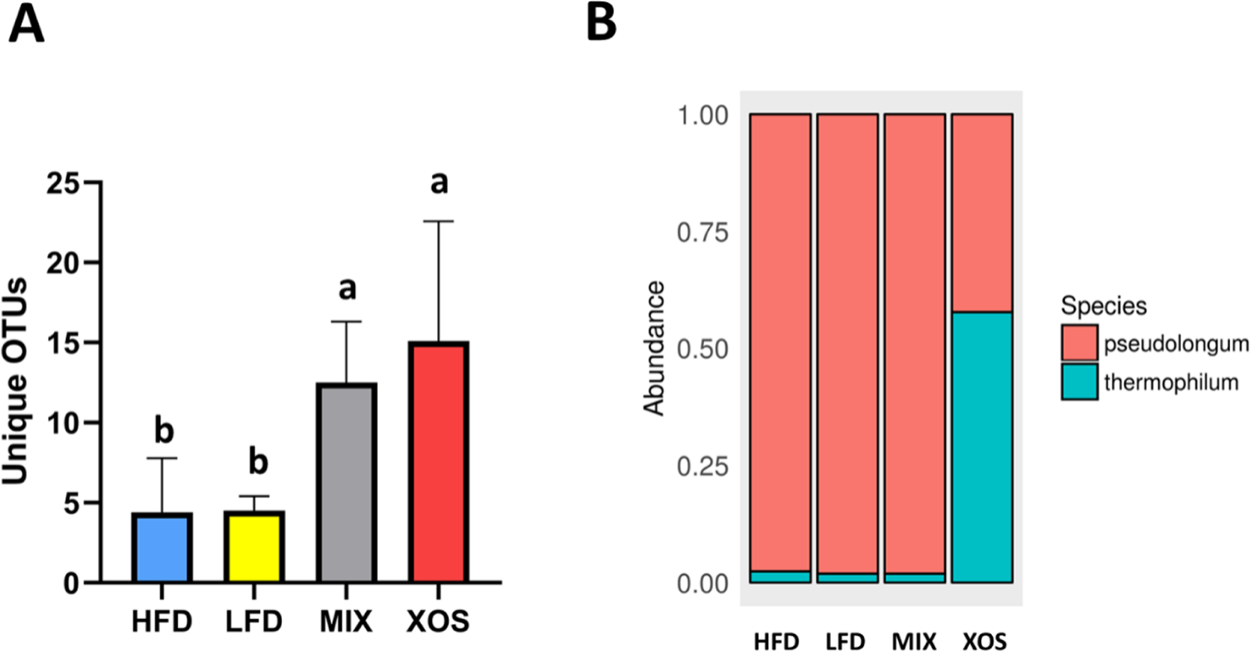
Unique OTUs by treatment and *Bifidobacterium* species by treatment. A: Total unique OTUs by treatment. Error bars represent standard deviation. Bars with different lowercase letters are statistically different from each other (*p* < 0.05). B: *Bifidobacterium* species by treatment as determined by NCBI blasting a subset of reads from each treatment (*n* = 100) that had been mapped to the genus *Bifidobacterium* as determined by the standard Qiime pipeline.

There were no strong visual differences in the taxonomic profiles between the different diets, at the higher taxonomic levels, but based on Qiime’s Permanova statistics, there was an overall statistical difference (*p* < 0.01). The number of unique OTUs in the treatments were for example significantly different (Figure 2a) despite an insignificant Shannon alpha diversity result (data not shown). These differences in results might be because the standard Qiime-based pipeline has a cut-off resolution at the genus level and therefore cannot easily detect treatment differences at the species level. Indeed, subsampling reads in each treatment (*n* = 100) mapping to *Bifidobacterium* and blasting them at NCBI showed that the majority of reads in XOS mapped to *Bifidobacterium thermophilum*, while the other treatments were dominated by *Bifidobacterium pseudolongum* (*p* < 0.05) (Figure 2b). Similarly, subsampling *Bacteroides* showed alterations in the relative abundance of several species, including *Bifidobacterium vulgatus* and *Bifidobacterium uniformis* in response to treatment (data not shown).

**Figure 3:**
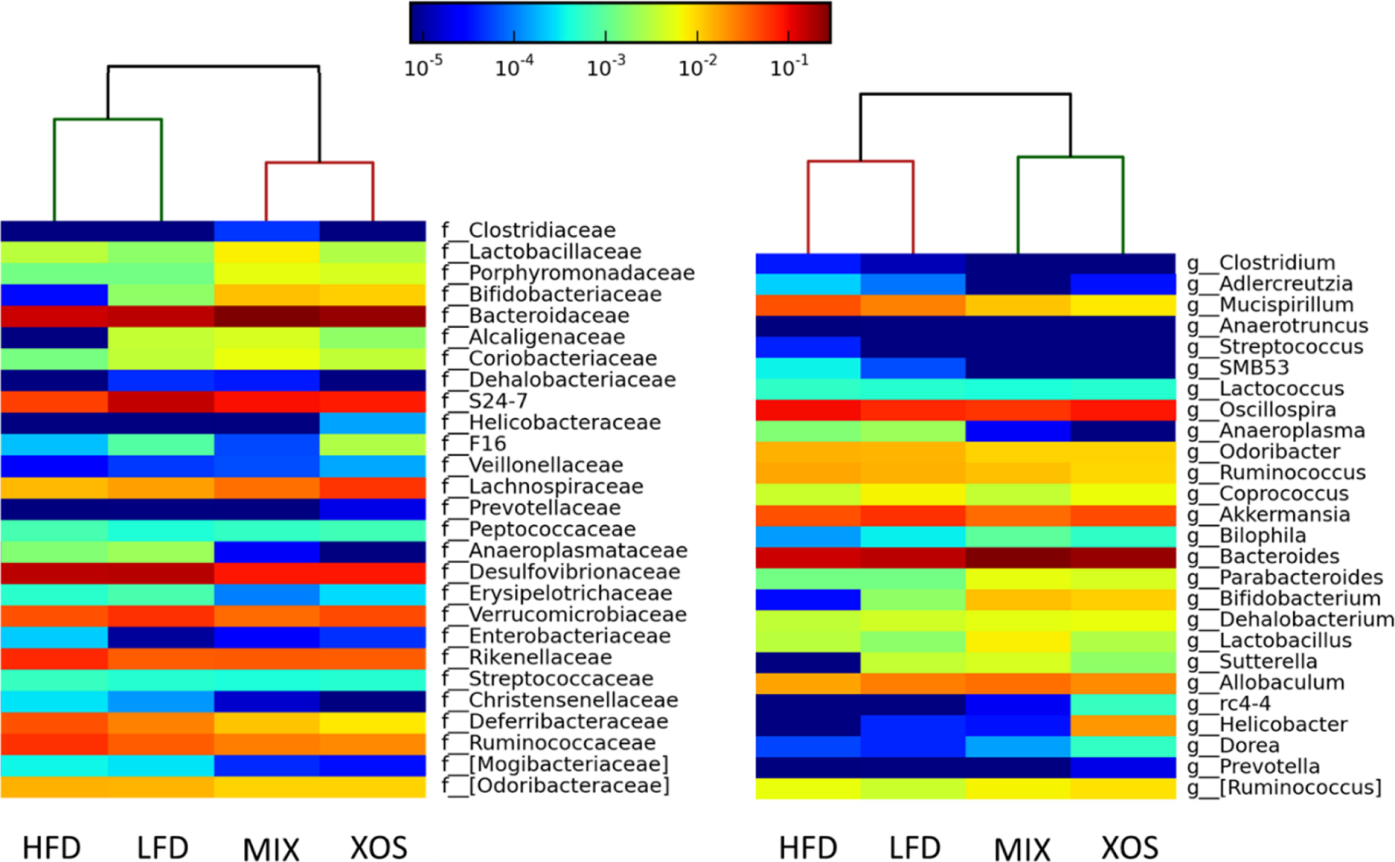
Metaphlan heat maps of families (left) and genera where possible (right) in each of the four treatments. Color based on the relative abundance scale at the top. The phylogenetic tree at the top separated the four treatments into two groups, a MIX + XOS group and an HFD + LFD group.

Visual differences were evident at the family and genus levels as shown in a Metaphlan heat map (Figure 3). The phylogenetic analysis associated with the heat map also showed an initial evidence that XOS and MIX treatments had more similar microbiomes relative to the HFD and LFD controls. Several bacterial genera were significantly different in the various treatments (*p* ≤ 0.05). Most notable was an increase in *Bacteroides*, *Bifidobacterium*, *Dehalobacterium*, and *Parabacteroides* in the cecum of mice fed MIX and XOS. XOS also had elevated levels of *Lachnospiraceae* and *Helicobacter*. Bacteria associated with HFD were the genera *Mucispirillum*, *Adlercreutzia*, *Oscillospira*, and the family *Rikenellaceae*. The undescribed genus S24-7 had a strong association with the LFD treatment.

**Figure 4:**
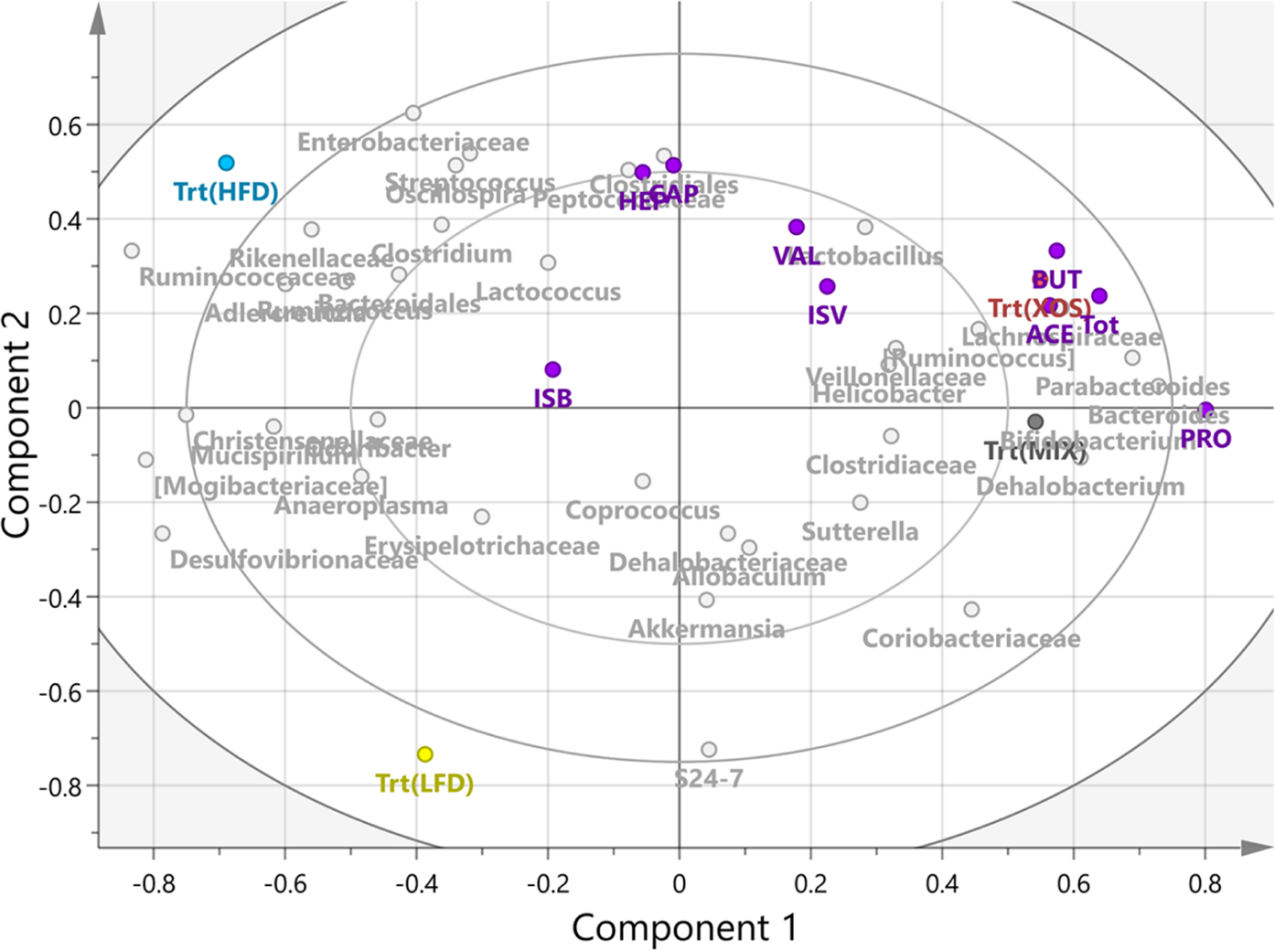
Multivariate analysis of the four treatments, nine SCFA biomarkers, and the cecal bacteria associated with each of the samples (*n* = 45). Calculated as a partial-least-squares (PLS) loading scatter plot colored according to variable ID and plotted without the observation variables visible. Plotted using Simca (v. 15.0.2, Umetrics Sweden). SCFA colored purple. The explained variance from SIMCA was reported as R2X[1] = 0.23, R2X[2] = 0.11.

Joining all the results in a Partial Least Squares bi-plot with the bacterial taxa and treatments as loading variables confirmed that total SCFAs, butyric acid, and acetic acid were strongly associated with the XOS treatment, while MIX was more closely associated with propionic acid (Figure 4). Superimposed on these relations were the bacterial taxa, which again showed the strong relationships described above and highlighted the differences of the dietary fiber composition between XOS and MIX. The main difference was that MIX was actually better correlated with *Bacteroides*, *Bifidobacterium*, *Dehalobacterium*, and propionic acid, while XOS was more strongly associated with butyric acid, *Parabacteroides*, and a number of other bacteria not found to be significant in the analyses described above. The bi-plot also revealed that MIX was highly negatively associated with HFD and its associated bacteria, which included a number of bacterial families, including the *Ruminococcaceae*, *Enterobacteriaceae*, and *Rikenellaceae* (Figure 4). The LFD treatment was surprisingly devoid of strong co-associations and was negatively associated with XOS and its co-associating bacteria.

**Figure 5:**
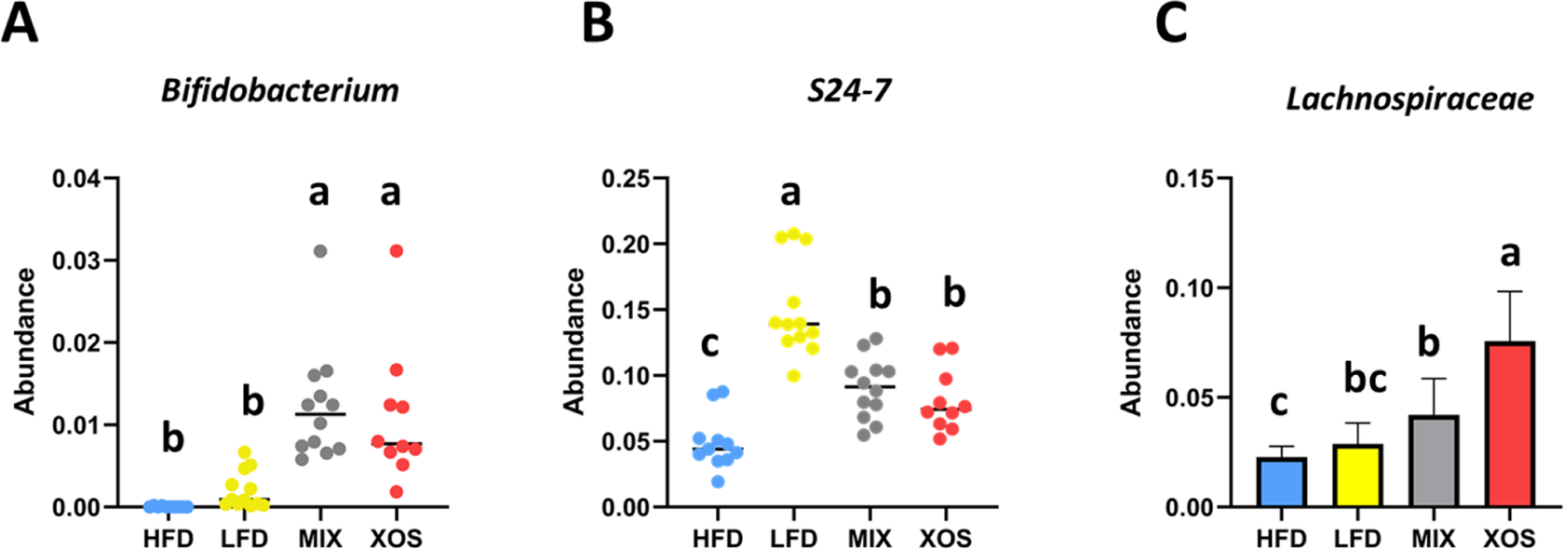
Abundance of the genus *Bifidobacterium* (A), the family S24-7 (*Muribaculaceae*) (B), and the family *Lachnospiraceae* (C) in the four treatments. The barchart presents the average of normally distributed data, while the boxplots present the median of non-normal data. Error bars represent the standard deviation. Bars with different lowercase letters are statistically different from each other (*p* < 0.05).

The abundance data on *Bifidobacterium*, S24-7, and *Lachnospiraceae* were analyzed in more detail. *Bifidobacteria* were significantly more abundant in the MIX and XOS groups than in the HFD and LFD control groups (Figure 5a). The abundance of S24-7 was dramatically lower in the HFD group compared to the LFD group, whereas the abundance in the MIX and XOS groups was in between these extremes (Figure 5b). The abundance of *Lachnospiraceae* was low in the HFD group and significantly higher in the MIX group and even more so in the XOS group (Figure 5c).

## Discussion

The HFD caused a considerable increase in weight gain compared to the LFD, as expected. The weight gain was accompanied with increased blood glucose and insulin levels. Although no significant differences in cecal SCFAs were observed between LFD and HFD control, the fat content of the diet had a substantial effect on the microbiota composition. This could be due to an increase in intermediary organic acids such as succinic and lactic acid.^4^ Interestingly, the family S24-7, (also known as *Muribaculaceae*) was lower (*P* < 0.05) in the HFD group compared to the LFD group. *Muribaculacae* is the major family in the gut microbiota of healthy mice.^15,16^ This family was observed to increase dramatically in treatment-induced remission in experimental colitis in mice.^17^ Our study thus agrees with the previous observations that *Muribaculacae* in the gut microbiota of mice is associated with good health although mechanistic explanations are still lacking. Interestingly, XOS and MIX diets increased *Muribaculacae* compared to HFD, thus indicating a positive effect.

XOS and MIX diets caused increased cecum weights due to the fact the dietary fiber in the diet reaches this part. Since these diets contain highly fermentable fibers, the increased cecal weights is most probably due to an increased abundance of bacteria and the high concentration of SCFAs in this part of the gastrointestinal tract and perhaps to some extent also small amounts of unfermented fiber that can bind water.

The different types of indigestible carbohydrates in the MIX diet were probably utilized to different extents by different organisms of the gut microbiota. However, surprisingly, the diversity in the microbiota was not significantly higher in the MIX group compared to the XOS group. Among the microbiota components identified, the increase in *Bifidobacterium* and *Lachnospiraceae* in the XOS and MIX groups is of special interest and will be further discussed below.

### *Bifidobacterium*

The results of this study agree with previously published data showing stimulation of *Bifidobacteria* by XOS. Within *Bifidobacterium*, the ability to utilize XOS and AXOS is strain-dependent.^18^ When the growth of several bacterial strains on various potential prebiotics was evaluated, it was found that XOS was more selective in stimulating certain *Bifidobacteria* than more established prebiotics such as galacto-oligosaccharides and fructo-oligosaccharides, which were used by a larger group of bacteria.^19^ In a colon model, it was found that XOS was especially efficient in stimulating the growth of *Bifidobacterium lactis*.^20^

In one mouse study, XOS was shown to stimulate *Bifidobacterium* throughout the intestine.^21^ Similarly, a significant increase in cecal *Bifidobacterium* was observed in mice on an HFD when fed a rye bran-derived product rich in XOS.^10^ Furthermore, stimulation of *Bifidobacterium* by dietary XOS has been demonstrated in a few human studies.^22−24^ It was noted that only *bifidobacteria* but not lactobacilli were stimulated.^23^ In addition, improvement in plasma lipids, modulation of markers of immune function, and increased participant-reported vitality and happiness were reported after XOS intake.^24^

The *Bifidobacterium* species identified in this study were *B. pseudolongum* and *B. thermophilum*. Phylogenomic analysis of the genome sequences of 60 *B. pseudolongum* strains has revealed that *B. pseudolongum* subsp. *globosum* and *B. pseudolongum* subsp. *pseudolongum* may represent two distinct bifidobacterial species.^25^*B. thermophilum* has been isolated from human feces and has been evaluated as a potential probiotic. A previous study showed that *B. pseudolongum* was a dominating species in the mouse microbiota, and after feeding with fructo-oligosaccharides, it became almost the sole bifidobacterial species (>95%).^26^ In the present study, *B. pseudolongum* was the dominating species in all groups with the exception of the XOS group, in which *B. thermophilum* accounted for more than 50% of the bifidobacteria. *B. pseudolongum* is widely distributed in the gut of mammals. Significant strain-dependent variations may, however, occur, for example, regarding growth on complex carbon sources such as XOS.^27^

### Predicted and Observed XOS Catabolism of *B. thermophilum* and *B. pseudolongum* sps

Growth studies using xylan and XOS involving *B. pseudolongum* isolated from human feces indicated that this strain cannot grow on xylan but can grow on and utilize XOS.^28^ The presence of β-xylosidase in the microbial cultures was observed along with formation of SCFAs during growth on XOS. *B. pseudolongum* subsp. *globosum* AGR 2145 have been shown to utilize XOS and this capacity involves a gene cluster encoding, for example, putative GH43 enzymes and a sugar-binding protein.^29^

Very limited information is available on *B. thermophilum* regarding growth and utilization of xylan and xylo-oligosaccharides. Rivière et al.^27^ showed that *B. thermophilum* exhibits only poor growth on XOS and AXOS and only one putative enzyme related to conversion of these carbon sources is encoded by its genome (strain RBL67) according to the carbohydrate active enzymes database (CAZy; http://www.cazy.org). Also, for this species it cannot be ruled out that significant strain variations may occur, which could be an explanation for the observed increased abundancy using the XOS diet.

### Predicted and Observed Catabolism of other MIX Components of *B. thermophilum* and *B. pseudolongum* sps

It is interesting that *B. thermophilum* was reduced in the MIX group. This suggests that the extra MIX components disfavored *B. thermophilum* compared to *B. pseudolongum*. This is perhaps less likely to be due to oligofructose or inulin since *B. thermophilum* has been shown to ferment these glycans *in vitro* and to produce associated hydrolase(s).^30^ In accordance with our data, the abundancy of *Bifidobacterium pseudologum* increased when mice were fed a diet with oligofructose,^31^ but *B. thermophilum* was not discussed in the previous study. The MIX diet also contains partially hydrolyzed guar gum (galactomannan), which could possibly favor *B. pseudolongum*. Animal model studies have indicated that both guar gum^32^ and mannan-oligosaccharides^33,34^ can positively influence the abundance of *B. pseudolongum* but as it appears, similar information is lacking for *B. thermophilum*. The utilization of galactomanno-oligosaccharides requires hydrolases that attack both galactose and mannose units, that is, α-galactosidase and β-mannanase or β-mannosidase, which are co-expressed by some gut bacteria.^35^*B. pseudolongum* has been reported to produce α-galactosidase.^36^ However, to the best of our knowledge, β-mannanase or β-mannosidase activity has not been reported for *B. pseudolongum* or *B. thermophilum* nor has α-galactosidase activity been described for the latter. Annotated genome data (in CAZy), however, support differences between the species as the genomes of *B. pseudolongum* (strains PV8-2 and UMB-MBP-01) encode a β-mannosidase and number of α-galactosidases, while the genome of *B. thermophilum* RBL67 only encodes a single α-galactosidase. Provided that the *B. pseudolongum* genes express functional enzymes, galactomannan could be converted to mono-sugars, while *B. thermophilum* RBL67 lack known hydrolases for galactomannan backbone degradation. Metagenomic studies would certainly be useful to further investigate the catabolism of the potential prebiotics.

### Butyric Acid Formation

Butyric acid has been shown to have several beneficial effects, including being an excellent nutrient for epithelial cells and having immune-modulating and anti-inflammatory effects.^37,38^ The MIX diet and especially the XOS diet was effective in causing formation of butyric acid and it is therefore of interest to evaluate which bacteria were involved in this process. An obvious candidate is the family *Lachnospiraceae*, which is common in the gut of mammals, especially humans, mice, and cows, but otherwise relatively rare.^15,16^ The *Lachnospiraceae* family includes known butyric acid producers, such as *Roseburia* and *Eubacterium*.

Both the MIX and XOS diets contain soluble fibers in the form of partially hydrolyzed xylan and mannan. Long-chain xylans and mannans can be cleaved by extracellular endo-xylanases and endo-mannanases to oligosaccharides. XOS and manno-oligosaccharides (MOS) of varying lengths can be taken up by butyrate-producing bacteria. Transporter systems in *Roseburia intestinalis* have been thoroughly characterized in the context of XOS and MOS uptake.^39,40^ The transporter systems can function with different lengths of oligosaccharides but is most efficient for degree-polymerization (DP) 4 and DP5. Other organisms with different transporters may be more efficient in taking up longer or shorter XOS. Once inside the bacterial cells, the oligosaccharides can be further degraded and eventually converted to butyric acid. The most common carbohydrate-based pathways involve the enzymes butyryl-CoA:acetate-CoA transferase or butyrate kinase.^37,41^

It has been shown in previous studies that arabinoxylan and partially degraded arabinoxylan can stimulate the growth of *Lachnospiraceae* and increase the formation of butyric acid. Enzymatically degraded arabinoxylan was efficient in promoting increased weight gain in broilers and the proposed mechanism was via stimulation of butyric acid producing *Lachnospiraceae* and *Ruminococcaceae*, which were found to increase in the ceca of the young broilers.^42^ Similarly, in humanized rats, long-chain arabinoxylan in the diet caused a significant increase in cecal content of *Eubacterium rectale*-like and *R. intestinalis*-like bacteria as well as a significant increase in butyric acid formation.^43^

The abundance of *Bifidobacterium* and the genus associated with *Lachnospiraceae* in the cecum of mice fed with XOS is consistent with the bifidogenic and butyrogenic effect of XOS as suggested by Riviere et al^18^ Cross-feeding mechanisms in which acetate, for example, produced by bifidobacteria are fed to butyrate producers (e.g., *Lachnospiraceae*) are key elements that favor the co-existence of these strains in the same ecological niche.^44^ Cross-feeding between different organisms in the gut microbiota occurs to a large extent. Concerning formation of butyric acid, cross-feeding with lactate or succinate as intermediary metabolites is, in addition, of great importance.^41^

In conclusion, XOS was shown to be a promising prebiotic candidate. The butyric acid content in the cecum increased significantly, possibly caused by stimulation of bacteria of the family *Lachnospiraceae*. A tendency of reduced weight gain was observed. In another study, up to 10% XOS was included in the HFD of mice and this significantly decreased the weight gain.^45^ The fiber diversity provided in our MIX diet did not provide any obvious advantages compared to the XOS diet, neither concerning physiological effects nor concerning increased diversity or improved composition of the gut microbiota.

## Supporting Information Available

Diet compositions (PDF)
