# Supplementary material for: Xylooligosaccharides Increase Bifidobacteria and Lachnospiraceae in Mice on a High-Fat Diet, with a Concomitant Increase in Short-Chain Fatty Acids, Especially Butyric Acid
Source: J Agric Food Chem. 2021 Mar 16;69(12):3617–25. doi: 10.1021/acs.jafc.0c06279 (PMC8041301; doi:10.1021/acs.jafc.0c06279)
Supplement: Supplementary file 1 — jf0c06279_si_001.pdf [file jf0c06279_si_001.pdf]

Table S1. Diet Composition. The Diets Are Formulated to Have Matched Macronutrient Composition by Energy. Diets Manufactured by Research Diets, NB, USA

	HFD Ctrl		LFD Ctrl		MIX		XOS	
**Ingredient (g/kg)**								
Casein		253		184		252		252
L-Cystine		3.8		2.8		3.8		3.8
Wheat starch		87.1		517.2		86.7		86.8
Maltodextrin 10		158		114		158		158
Sucrose		3.4		2.5		0		3.4
Cellulose, BW200		80.0		80.0		0		0
Soybean Oil		31.6		23.0		31.5		31.5
Lard		310		23.0		309		309
Minerals Mix S10026		12.7		9.2		12.6		12.6
DiCalcium Phosphate		16.5		11.9		16.4		16.4
Calcium Carbonate		7.0		5.1		6.9		6.9
Potassium Citrate, 1 H2O		20.9		15.2		20.8		20.8
Vitamin Mix V10001		12.7		9.2		12.6		12.6
Xylooligosaccharides		0		0		29.3		83.3
Maritene (guar gum)		0		0		29.3		0
Orafti P95 (oligofructose)		0		0		14.3		0
Orafti GR (inulin)		0		0		14.3		0
**Total (g)**		**1000.0**		**1000.0**		**1000.0**		**1000.0**
**Kcal/g**		**5.2**		**3.7**		**5.1**		**5.1**
	g%	kcal%	g%	kcal%	g%	kcal%	g%	kcal%
**Protein**	25.7	20	18.6	20	25.6	20	25.6	20
**Carbohydrate**	26.1	20	64.4	69	26.0	20	26.1	20
**Fat**	34.2	60	4.6	11	34.0	60	34.1	60
**Fiber**	8.0	0	8.0	0	8.0	0	8.0	0
